# Toward Quantitative Electrodeposition via In Situ Liquid Phase Transmission Electron Microscopy: Studying Electroplated Zinc Using Basic Image Processing and 4D STEM

**DOI:** 10.1002/smtd.202400081

**Published:** 2024-04-30

**Authors:** Junbeom Park, Sarmila Dutta, Hongyu Sun, Janghyun Jo, Pranav Karanth, Dieter Weber, Amir H. Tavabi, Yasin Emre Durmus, Krzysztof Dzieciol, Eva Jodat, André Karl, Hans Kungl, Yevheniy Pivak, H. Hugo Pérez Garza, Chandramohan George, Joachim Mayer, Rafal E. Dunin‐Borkowski, Shibabrata Basak, Rüdiger‐A Eichel

**Affiliations:** ^1^ Institute of Energy and Climate Research Fundamental Electrochemistry (IEK‐9) Forschungszentrum Jülich GmbH 52425 Jülich Germany; ^2^ DENSsolutions B.V. Informaticalaan 12 Delft 2628 ZD Netherlands; ^3^ Ernst Ruska‐Centre for Microscopy and Spectroscopy with Electrons and Peter Grünberg Institute Forschungszentrum Jülich GmbH 52425 Jülich Germany; ^4^ Department of Radiation Science and Technology Delft University of Technology Mekelweg 15 Delft 2629JB Netherlands; ^5^ Dyson School of Design Engineering Imperial College London London SW7 2AZ UK; ^6^ Central Facility for Electron Microscopy (GFE) RWTH Aachen University 52074 Aachen Germany; ^7^ Institute of Physical Chemistry RWTH Aachen University 52074 Aachen Germany

**Keywords:** 4D STEM, dendrites, image processing, in situ/operando, LPTEM

## Abstract

High energy density electrochemical systems such as metal batteries suffer from uncontrollable dendrite growth on cycling, which can severely compromise battery safety and longevity. This originates from the thermodynamic preference of metal nucleation on electrode surfaces, where obtaining the crucial information on metal deposits in terms of crystal orientation, plated volume, and growth rate is very challenging. In situ liquid phase transmission electron microscopy (LPTEM) is a promising technique to visualize and understand electrodeposition processes, however a detailed quantification of which presents significant difficulties. Here by performing Zn electroplating and analyzing the data via basic image processing, this work not only sheds new light on the dendrite growth mechanism but also demonstrates a workflow showcasing how dendritic deposition can be visualized with volumetric and growth rate information. These results along with additionally corroborated 4D STEM analysis take steps to access information on the crystallographic orientation of the grown Zn nucleates and toward live quantification of in situ electrodeposition processes.

## Graphical abstract

This manuscript presents a workflow that allows for the visualization of 3D electrodeposition via in situ liquid phase transmission electron microscopy (LPTEM), advancing the study of dendirte growth spatiotemporally with information about their crystallographic orientation.

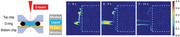

## 1 Introduction

Liquid phase transmission electron microscopy (LPTEM) is an emerging technique to observe nanoscale phenomena in liquid phase.^[^ ^1^, ^2^, ^3^ ^]^ While low vapor pressure liquid like ionic liquid^[^ ^4^, ^5^ ^]^ can be introduced directly in the TEM vacuum, the high vapor pressure liquids need to be sandwiched within electron transparent materials such as graphene^[^ ^6^ ^]^ or ultrathin silicon nitride film.^[^ ^7^ ^]^ Graphene liquid cell has advantages of high spatial resolution, stability against electron beam damage due to graphene's high thermal and electrical conductivity, while micro‐electro‐mechanical system (MEMS)‐based cell with electron transparent silicon nitride windows has advantages of functionality such as liquid flowing, heating and biasing. With these, LPTEM has enabled the investigation of various phenomena such as nanoparticle growth and electrochemical reaction.^[^ ^8^, ^9^, ^10^ ^]^

Metal plating and stripping is one of the important phenomena in the context of electrochemical energy storage because it strongly influences the efficiency, longevity and safety of batteries. Dendrite growth during cycling is one severe problem that can jeopardize durability and safety of energy storage devices and a considerable amount of effort has been made to inhibit the dendrite growth.^[^ ^11^ ^]^ One example case is aqueous zinc (Zn)‐based battery chemistry, including redox flow battery,^[^ ^11^ ^]^ which has recently generated significant interest as an alternative battery technology for stationary applications beyond Li‐ion batteries due to its high volumetric capacity, low cost, safety, and abundance (Zn metal). Despite these advantages, alongside electrode passivation, anode shape change,^[^ ^12^ ^]^ and H_2_ evolution, the problems of Zn dendrite growth^[^ ^13^ ^]^ cause premature battery failure and safety hazards, severely limiting the progress and further commercial exploitation of aqueous Zn batteries.^[^ ^14^ ^]^ A wide range of mitigations, including the application of organic polymers/co‐solvent stabilizing Zn deposits,^[^ ^15^ ^]^ nanocarbon‐based scaffolds containing deposits,^[^ ^16^ ^]^ self‐assembled monolayers with electrostatic attraction preventing dendrites,^[^ ^17^ ^]^ hydrogel as protective films,^[^ ^18^ ^]^ deposition of Zn as coating and varying current densities,^[^ ^19^ ^]^ hexagonal patterns with specific crystal facets^[^ ^20^ ^]^ altering the direction of Zn growth and pulse deposition techniques^[^ ^21^ ^]^ and different electrolyte combinations have been proposed. However, the formation of Zn dendrites is thermodynamically more favorable, and their nucleation and growth are a complex process.^[^ ^22^, ^23^ ^]^ One of most difficult tasks is to control metal plating and stripping events reversibly and spatiotemporally under actual electrochemical conditions across different length scales. Thus, direct visualization of dendrites under operando conditions can lead to in‐depth understanding and the development of the most effective mitigation routes toward achieving a compact plating and smooth stripping of Zn during battery cycling, which is a prerequisite for battery safety, longevity, and viability.^[^ ^24^ ^]^

Among the many Operando/in situ studies developing insights into these processes using techniques such as XCT,^[^ ^25^, ^26^, ^27^ ^]^ optical microscopy^[^ ^28^, ^29^ ^]^ and X‐ray‐based imaging,^[^ ^30^ ^]^ the use of TEM can be very effective in enabling a more detailed understanding of Zn plating/stripping. The unique advantage of TEM‐based studies is the possibility to locally probe and visualize the mechanistic processes governing the dendrite growth up to the sub‐nm scale, together with chemical information with high spatial and temporal resolutions, which are basically inaccessible via other methods.^[^ ^31^ ^]^ Among many interesting studies, Sasaki et al.^[^ ^32^ ^]^ have reported using a MEMS‐based liquid cell with Pt‐based electrodes, where Zn dendrites grow from regions close to the roots of dendrites, which was related to a concentration gradient between the electrode's surface and its surroundings for the observed difference in Zn dissolution. Li et al.^[^ ^33^ ^]^ also have used liquid phase TEM to investigate the formation of Zn dendrites, where the effect of applied current and electrolyte flow rate on the initial stages of dendrites was characterized to be a diffusion‐limited growth, in which a square root relationship between the time and dendrites growth (mostly lateral direction) was observed. Huang et al.^[^ ^34^ ^]^ have reported the influence of cations and anions from electrolytes additives on the growth of Zn dendrites, where the suppression of dendrites was visualized. However, TEM studies are still lacking in probing the exact nature of metal deposits and their crystal domain orientation. Furthermore, the correlation between electrical stimuli and actual volume of Zn plating/stripping are some of the questions that need further elucidation. This requires new and simple methods to characterize how metal plating and stripping performs along with their correlative electrochemical data via concurrent image processing and data analysis via TEM. In this work, we have performed electro‐plating/stripping of Zn using a MEMS‐based liquid cell^[^ ^7^, ^35^, ^36^ ^]^ in STEM mode. Through image processed STEM movies complemented with 4D STEM analysis that aims toward the live processing, we have developed a workflow that allowed for detailed characterization of dendritic growth. This allows for the visualization of 3D electrodeposition at the nanoscale during in situ experiments, enabling the study in terms of dendrite growth across all three planes (XY, YZ, and XZ) and information about their crystallographic orientation.

## 2 Results and Discussion

### 2.1 Cyclic Voltammetry

**Figure 1:**
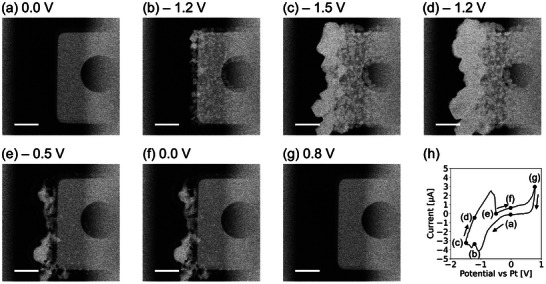
a–g) HAADF STEM images during Zn plating (a–d, at 0.0, −1.2, −1.5, and −1.2 V) and stripping (e–g, at −0.5, 0.0, and 0.8 V) process in 0.1 m ZnSO_4_ solution on the finger‐shaped Pt working electrode during the cyclic voltammetry. The scale bars in (a–g) are 2 µm. h) The corresponding Current versus Potential curve during CV. The black dots on the CV curve in (h) indicate the biasing condition at the corresponding STEM images shown in (a–g). The flow rate of the 0.1 m ZnSO_4_ was 1.7 µL min^−1^, and the potential scan rate was 0.1 V s^−1^.

In situ Zn plating/stripping experiment was performed using a commercial electrochemical TEM holder (Figure S1, Supporting Information). **Figure** **1**a‐g show the high‐angle annular dark field (HAADF) STEM images recorded at certain potentials during the plating and stripping of one of the fingers of the Pt working electrode (Figure S1, Supporting Information). Figure 1a shows the pristine Pt electrode before the Zn plating. As the potential sweeps to negative values, Zn plating starts to occur. Figure 1b shows the early stage growth of Zn plating that had occurred till −1.2 V. Amount of plated Zn continues to increase as the potential reaches the lowest value of −1.5 V (Figure 1c). The plated Zn continues to grow, reaching its maximum value as the scanning potential returns to −1.2 V (Figure 1d). The initial stripping starts as the current turns positive, and Figure 1e shows the initial stripping that had occurred till −0.5 V. An increase of the potential to 0 V (Figure 1f), has virtually no effect on Zn stripping at least on this Pt finger as there is no obvious visual change between −0.5 (Figure 1e) and 0 V (Figure 1f). At the potential of 0.8 V (Figure 1g), the Zn is completely stripped from the Pt electrode, so it looked similar to pristine electrode (Figure 1a). Figure 1h shows the corresponding cyclic voltammetry (CV) during the experiment, with highlighting the potentials at which the STEM images of Figure 1a–g are obtained. It should be noted that only a part of the Pt working electrode (one finger) was in the field of view (Figure S1c, Supporting Information), meaning the Zn plating/stripping at other locations on the electrode couldn't be observed. For this reason, obtaining a direct quantitative correlation between the amounts of plated Zn with the electrochemical data is practically very difficult. Suitable reference and working electrode are an important consideration here. Due to the compatibility issues with LPTEM, MEMS chips and processing, Pt microelectrodes are used here as pseudo reference, however, to get a better sense of the cell potentials, we have performed three‐electrode beaker cell tests comparing Pt and Ag/AgCl as reference electrode (Figure S2, Supporting Information). Approaches leading to a direct correlation of the potential with respect to a standard reference electrode should also further be investigated. On the other hand, for proper correlation analysis, it's necessary to have the complete working electrode in the field of view. However, the increased possibility to loss of connection with the working electrode due to electrochemically induced bubble formation should be kept in mind. Replacing the material of the working electrode with other conductive materials with higher hydrogen evolution potential can help reduce hydrogen evolution. We are currently exploring carbon and titanium nitride‐based electrodes for this purpose.^[^ ^37^ ^]^

**Figure 2:**
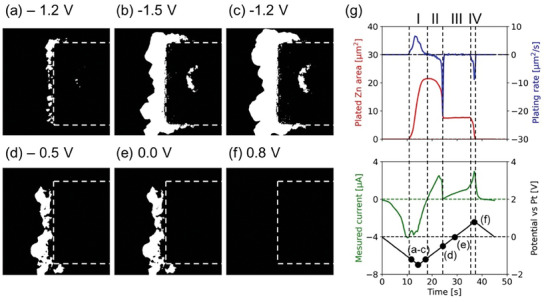
a–f) Processed images of plated Zn while plating (a–c, at −1.2, −1.5, and −1.2 V) and stripping (d–f, at −0.5, 0.0, and 0.8 V). The image processing was performed by subtracting the reference STEM image (only the Pt electrode) from the target STEM image after Gaussian filtering on each image and thresholding the subtracted image (Figure S3, Supporting Information). The dotted box on each processed image represents the Pt electrode area. g) The graphs about the plated Zn area, plating rate, the measured current, and the applied potential versus time during the CV. The spots in (g) indicate the biasing condition at the corresponding STEM images shown in (a–f).

Image processing has been applied to the recorded STEM images to extract information about the details and the amount of the plated and stripped Zn during the CV. **Figure** **2**a–f shows the processed images of Zn plating/stripping at different potentials. Compared to the original HAADF STEM images (Figure 1b–g), these processed images show purely the Zn being plated and stripped during the CV cycle. From these binarized images, the plated Zn area and plating rate, can be estimated at each point by counting the number of pixels (Figure 2g). By analyzing the plated Zn area, it can be concluded that the Zn plating is a single step process (only at I) while the Zn stripping consists of two steps (II at negative potentials and IV at positive potential). The graph has a flat area (III) where the potential increase leads to no Zn stripping. The complete Zn plating/stripping process during the CV cycle is shown in Movie S1, Supporting Information.

In principle, the intensity of the HAADF STEM image is strongly dependent on the average atomic number of the sample and thus called Z or mass‐thickness contrast imaging. As the Zn was electroplated without additives, the intensity value of the HAADF STEM image would be proportional to the thickness of the platted Zn. Figure S4a–f, Supporting Information are the reconstructed images corresponding to the original HAADF STEM images (Figure 1b–g), showing morphological information of Zn plating/stripping at different potentials obtained after Gaussian filtering and subtraction. It should be noted that the intensity on the Pt electrode area was almost saturated (see Figure S5, Supporting Information), causing the plating on top of the Pt electrode region to be slightly underestimated. But the Zn plating outside the Pt electrode area can be clearly visualized, and this 3D projection can give a qualitative insight into Zn plating phenomena.

### 2.2 Dendrite Growth

**Figure 3:**
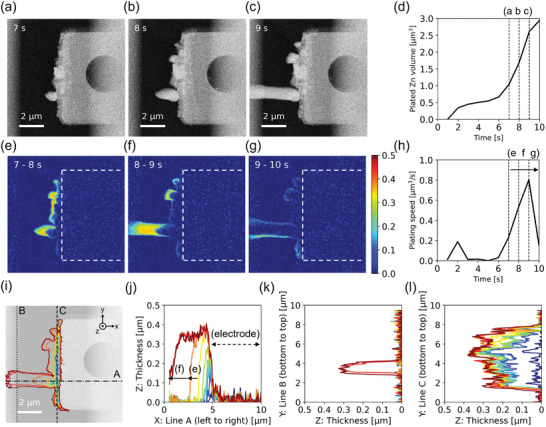
Investigation of Zn dendritic growth during plating in static 0.1 m ZnSO_4_. a–c) STEM images obtained at seventh, eighth, and ninth seconds. d) The plated Zn volume versus time. e–g) Processed images show the Zn dendritic growth between 7–8, 8–9, and 9–10 s. A color map on (g) represents deposit thickness at eighth (e), ninth (f), and 10^th^ (g) second. The white dotted rectangle in (e–g) represents the Pt electrode. h) The plating speed of Zn versus time. i–l) Tracking profiles of Zn dendritic growth: i) along X‐Y plane, top‐view of the dendrite, j) along X‐Z plane, thickness versus horizontal dash‐dot line A in (i), k) along Y‐Z plane, thickness versus vertical dotted line B in (i), and (l) along Y‐Z plane, thickness versus vertical dashed line C in (i). The profile's color indicates the time from 1s (blue) to 10 s (red). The background image in (i) is pristine Pt electrode.

Dendritic growth is an important topic in many electrochemical applications, as the high aspect ratio of the dendrite can easily cause a short circuit. The Zn dendritic growth was studied using chronopotentiometry (−5 µA current applied for 10 s) with no flow of electrolyte. **Figure** **3**a–c shows the HAADF STEM images of plated Zn with µm‐size dendrites grown within 10 s (Movie S2, Supporting Information). To overcome the saturation issue on STEM image intensity at the Pt electrode area, the intensity‐thickness calibration has been done by assuming a semi‐cylindrical shape of the Zn dendrite (Figure S6, Supporting Information). The plated volume of Zn was then calculated by multiplying the area with the estimated thickness for the grown Zn. As can be seen from Figure 3d, the dendrite grows  rapidly. In order to investigate this fast change, the Zn plating information of each second was extracted. Figure 3e–g are the processed images that directly visualize how Zn dendrite grows at each second. The colored Zn dendritic growth showed horizontal symmetric growth to the center line of the dendrite (Figure 3e,f). After the drastic dendritic growth, shallow thickening was observed (Figure 3g). Figure 3h shows the plating speed of Zn [µm^3^ s^−1^] calculated by summation of pixel intensity of the processed image. At 2 s, a little plated Zn is recognized, where initiation at several positions occurs. After a few seconds of a sporadic growth phase, drastic dendritic growth is recognized after 6 s, possibly due to mass transport limitation, and ends at 10 s. These dynamics can be observed from Movie S2, Supporting Information.

For characterizing dendritic growth, it is important to distinguish between the growth along XY, YZ, and XZ plane separately. Figure 3i,j shows the morphological evolution of dendrites at each second along these planes. Figure 3i shows that plated Zn along the XY plane is mostly occurred at the edge or outside of the Pt electrode but not on top of the electrode. Figure 3j shows that the Zn dendrite along XZ plane propagates toward left side mainly within 2 s (yellow, orange, and red). Figure 3k shows the sudden appearance of dendrite and no other transition along YZ plane far from the Pt electrode. Figure 3l shows that Zn was plated evenly along YZ plane all over the electrode's edge except for initial quick growth. Thus, this process allows us to detect plating phenomena along different planes to shed light on the dendrite growth process.

### 2.3 4D STEM

**Figure 4:**
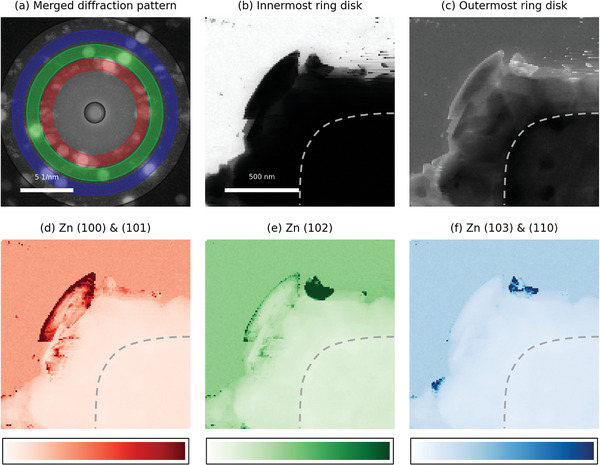
Virtual ring detector method. a) Merged electron diffraction patterns from the whole image area with Grey (innermost and outermost), Red (Zn (100) & (101)), Green (Zn (102)), and Blue (Zn (103) & (110)) ring masks. b–f) Reconstructed images from diffraction patterns with corresponding ring masks. The dotted shape in images (b–f) represents the Pt working electrode. Color bars in (d–f) represent relative intensity along each image.

**Figure 5:**
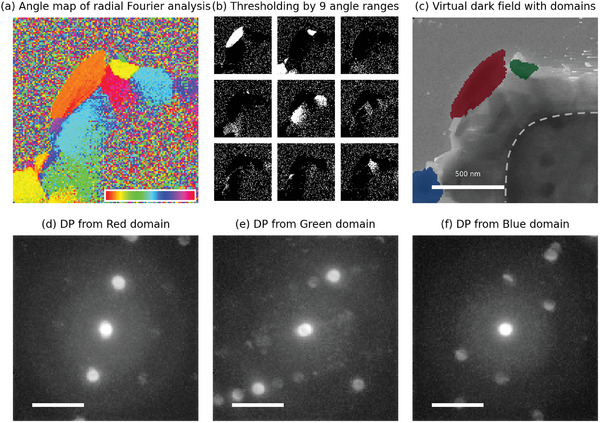
Radial Fourier analysis method. a) Angle map image after radial Fourier analysis (second order values between 4.1 and 7.8 nm^−1^). The inset color bar represents converted angle values between 0 and 360°. (b) Thresholded images by nine different angle ranges (360/9 = 40°). The white areas from each thresholded image are extracted as domain candidates. From each extracted domain candidates, electron diffraction patterns were reconstructed and the top 3 domains were determined by standard deviation, while clearly recognizable electron diffraction patterns show high contrast (high standard deviation). c) Virtual dark field image of plated Zn with top 3 mapped domains. The dotted shape in (c) represents the Pt working electrode. d–f) Reconstructed electron diffraction patterns dedicated to each top 3 mapped region in (c). The scale bars in (d–f) are 5 nm^−1^.

In principle, dendrite growth prefers a certain crystallographic orientation, so investigating the dendrite growth with crystallographic information is essential. 4D STEM^[^ ^38^ ^]^ is an electron microscopy technique capable of recording a 2D electron diffraction pattern at each pixel position in a 2D image, which can be used for virtual imaging, orientation/strain mapping, and differential phase contrast. Since the inception of 4DSTEM technique, a number of studies have utilized 4D STEM analysis to extract orientation information. Recently, a few LPTEM studies, both in graphene liquid cells and MEMS‐based devices, showcase the effectiveness of 4DSTEM.^[^ ^39^, ^40^, ^41^, ^42^ ^]^ Nevertheless, there are no studies yet that focus on obtaining orientation of dendritic growth. Further, analysis of the 4D STEM dataset is performed as post‐analysis due to its large size and complexity. However, for in situ TEM experiments, live processing of 4D STEM can be a powerful tool to determine the trend in real time. Here, open‐source platforms such as LiberTEM^[^ ^43^ ^]^ offer a promising solution. By enabling researchers to develop and implement custom workflows tailored for live analysis, LiberTEM can pave the way for future advancements in LPTEM. To explore the possibility of a fast analysis of 4D STEM, we performed image reconstruction for visualizing the orientation information of plated Zn by 1) virtual ring detector method (**Figure** **4**) and 2) radial Fourier analysis method (**Figure** **5**) with python and LiberTEM software. One of the prerequisites for obtaining good diffraction signal in LPTEM is thinner liquid layer. Some of the previous studies utilize electrochemically generated bubble to reduce liquid thickness at that particular position.^[^ ^40^ ^]^ However, given the limited control over the bubble(s), to perform 4D STEM analysis directly in the liquid phase, we reduced the liquid thickness to ≈100 nm by on‐chip flow coupled with the pressure‐based pump.^[^ ^36^ ^]^ This avoids the loss of diffraction information from plated Zn by scattering within the liquid.

The virtual ring detector method reconstructs the image based on the selected d‐spacing range, so the areas with strong intensity indicates the potential candidates of crystal domains corresponding d‐spacing direction. From the 4D STEM dataset of plated Zn, the merged electron diffraction pattern (Figure 4a) can be obtained. Each ring mask (grey, red, green, and blue) in Figure 4a represents the innermost & outermost disks, Zn (100) & (101), Zn (102), and Zn (103) & (110), respectively. Reconstructed images based on each ring mask (Figure 4b–f) indicate the strength of crystallinity toward corresponding in‐plane Zn d‐spacing at each pixel. In Figure 4b, the white area indicates the SiN window background area and the black area indicates the plated Zn and Pt electrode area. In Figure 4c, the overall morphology of plated Zn on Pt electrode is recognizable. In Figure 4d–f, the areas with high intensity can indicate the crystallographic domain candidates. Figure 4d has one big dark‐red area in the middle of the image. Similarly, Figure 4e has one dark green area next to the dark red area in Figure 4d. Two dark‐blue areas in Figure 4f were neglected in further discussion because one is overlapped with an area in Figure 4d and the other is too small to be a domain. Finally, across the Figure 4d–f, two domain candidates are found for further analysis such as recognizing the crystallographic orientation by matching with simulated electron diffraction pattern.

The radial Fourier analysis method reconstructs the image based on the representative angle (≈0–360°) of the diffraction pattern. An angle map can be obtained from the 4D STEM dataset of plated Zn using the radial Fourier analysis method with second order values between 4.1 and 7.8 nm^−1^ range (Figure 5a). As the pixels in the same crystal domain have the same orientation angle, the reconstructed images can visualize crystallographic domain candidates by thresholding with a narrow angle range (Figure 5b). The top 3 selected domains are displayed on a virtual dark field image (Figure 5c), and corresponding electron diffraction patterns are reconstructed (Figure 5d–f). The top 3 domains are selected among domain candidates based on the standard deviation value of reconstructed electron diffraction patterns (Figure S7, Supporting Information), matching closely with simulated electron diffraction patterns of [211], [110], and [101] zones (Figure S8, Supporting Information).

The results from each method are comparable. The candidate domain with Zn (100) & (101) ring detector (Figure 4d) matches the red domain (Figure 5c), and the candidate domain with Zn (102) and Zn (103) & (110) ring detectors (Figure 4e,f) match with green domain (Figure 5c). In the case of the virtual ring detector method, the algorithm is straightforward to visualize the potential crystallographic domains based on d‐spacing information enough to apply for live processing purposes, but the sensitivity of d‐spacing value is limited due to disk size (≈1 nm^−1^). On the other hand, in the case of the radial Fourier analysis method, the reconstructed map can show both determined domain map and corresponding electron diffraction patterns, but the algorithm is a little bit complicated multi‐step structures which may require high computing power to fulfill a live processing purpose. One remark is the possible misleading of radial Fourier analysis when the sample has similar diffraction patterns with different d‐spacing, because the radial Fourier analysis integrates the signal along each angle.

## 3 Conclusion

In conclusion, we have demonstrated a basic and effective image/data processing for obtaining in‐depth information about Zn plating from in situ liquid phase TEM. Firstly, this simple image processing based on background subtraction and denoising of STEM dataset allowed us to observe dendrite growth in X‐Y, Y‐Z and X‐Z planes, leading to volumetric information on dendrites. Secondly, the in situ reduction of electrolyte thickness in MEMS cell (LPTEM) allowed us to acquire high‐quality 4D STEM data, offering crystallographic information on dendrites. Furthermore, the rapidness and simplicity of 4D STEM data analysis method make it a promising candidate for live processing. The methods based on image processing and analysis we reported herein can be extended to other electrochemical systems (e.g., Li, Na, Mg etc.), for developing a comprehensive understanding of metal plating and stripping to advance battery technologies and electroplating techniques.

## 4 Experimental Section

### In Situ Zn Plating/Stripping Experiment

The in situ workflow consisted of the following steps: 1) preparing the 0.1 m ZnSO_4_solution, 2) assembling the in situ TEM holder, 3) checking the leak tightness of the holder, 4) inserting the assembled holder into TEM, 5) connecting tubings for liquid delivery and a biasing cable to the holder, 6) locating a region of interest and taking an image in a dry state, 7) flowing the prepared electrolyte solution, and 8) applying the potential (cyclic voltammetry between −1.5 and 0.8 V with 0.1 V s^−1^ scan rate, the potential was measured against Pt reference electrode) and capturing STEM image series. Electron beam dose rate of ≈100 e^−^/nm^2^s was used during the experiments.

### Image Processing

Related to Figure 2: To extract the data related to Zn plating, the STEM images were 1) applied the Gaussian filter to denoising, 2) subtracted the static background information (Pt electrode and SiN window) to identify only the plated Zn, and 3) binarized between the feature (white) and the background (black) using the equation below:

(1) $$P_{n}=\left\{G\left(I_{n}\right)-G\left(I_{0}\right)\right\}>T$$

Where *P_n_* is a processed image at time *n*, *G* is a Gaussian filter (sigma = 3), *I_n_* is a STEM image recorded at time *n*, *I_0_* is the initial STEM image recorded before the plating (Figure 1a) and T is a threshold value (Figure S3, Supporting Information).

Related to Figure 3: In order to investigate this fast change, the Zn plating information of each second was extracted using the following equation:

(2) $$Q_{n}=\left\{G\left(I_{n}\right)-G\left(I_{n-1}\right)\right\}$$

Where, *Q_n_* is a processed image depicting the amount of plated Zn occurred between (*n‐1)th* and *nth second*, *G* is a Gaussian filter (sigma = 3), and *I_n_* is a STEM image obtained at *nth second*.

## Conflict of Interest

The authors declare no conflict of interest.

## Supporting information

Supporting Information

Supplemental Movie 1

Supplemental Movie 2

## Data Availability

The data that support the findings of this study are available in the supplementary material of this article.
