# Supplementary material for: Toward Quantitative Electrodeposition via In Situ Liquid Phase Transmission Electron Microscopy: Studying Electroplated Zinc Using Basic Image Processing and 4D STEM
Source: Small Methods. 2024 Apr 30;8(12):2400081. doi: 10.1002/smtd.202400081 (PMC11672167; doi:10.1002/smtd.202400081)
Supplement: Supplementary file 1 — Supporting Information [file SMTD-8-2400081-s001.pdf]

small
methods

Supporting Information

for *Small Methods*, DOI 10.1002/smtd.202400081

Toward Quantitative Electrodeposition via In Situ Liquid Phase Transmission Electron Microscopy: Studying Electroplated Zinc Using Basic Image Processing and 4D STEM

Junbeom Park, Sarmila Dutta, Hongyu Sun, Janghyun Jo, Pranav Karanth, Dieter Weber, Amir H. Tavabi, Yasin Emre Durmus, Krzysztof Dzieciol, Eva Jodat, André Karl, Hans Kungl, Yevheniy Pivak, H. Hugo Pérez Garza, Chandramohan George, Joachim Mayer, Rafal E. Dunin-Borkowski, Shibabrata Basak and Rüdiger-A Eichel*

Supporting information

Towards Quantitative Electrodeposition via In-situ Liquid Phase Transmission Electron Microscopy: Studying Electroplated Zinc using Basic Image Processing and 4D STEM

Junbeom Park[1], Sarmila Dutta[1], Hongyu Sun[2], Janghyun Jo[3], Pranav Karanth[4], Dieter Weber[3], Amir H. Tavabi[3], Yasin Emre Durmus[1], Krzysztof Dzieciol[1], Eva Jodat[1], André Karl[1], Hans Kungl[1], Yevheniy Pivak[2], H. Hugo Pérez Garza[2], Chandramohan George[5], Joachim Mayer[3,6], Rafal E. Dunin-Borkowski[3], Shibabrata Basak[1,*], Rüdiger-A Eichel[1,7]

[1] Institute of Energy and Climate Research, Fundamental Electrochemistry (IEK-9), Forschungszentrum Jülich GmbH, 52425 Jülich, Germany

[2] DENSsolutions B.V., Informaticalaan 12, 2628 ZD Delft, The Netherlands

[3] Ernst Ruska-Centre for Microscopy and Spectroscopy with Electrons and Peter Grünberg Institute, Forschungszentrum Jülich GmbH, 52425 Jülich, Germany

[4] Department of Radiation Science and Technology, Delft University of Technology, Mekelweg 15, Delft, 2629JB, Netherlands

[5] Dyson School of Design Engineering, Imperial College London, SW7 2AZ, London, UK

[6] Central Facility for Electron Microscopy (GFE), RWTH Aachen University, 52074 Aachen, Germany

[7] Institute of Physical Chemistry, RWTH Aachen University, 52074 Aachen, Germany

*Corresponding Author: Shibabrata Basak (s.basak@fz-juelich.de)

Instruments and chemicals used in this work

- TEM (FEI Titan G2 60-300 HOLO)
- TEM (FEI Titan G2 80-200 ChemiSTEM) with 4D STEM detector (EMPAD)
- In situ TEM holder with liquid flow and biasing capabilities (Stream LB system, DENSsolutions)
- Potentiostat (PalmSens 4C)
- 0.1 M $ZnSO_4$ aqueous solution prepared with de-ionized water

The liquid phase in-situ TEM holder

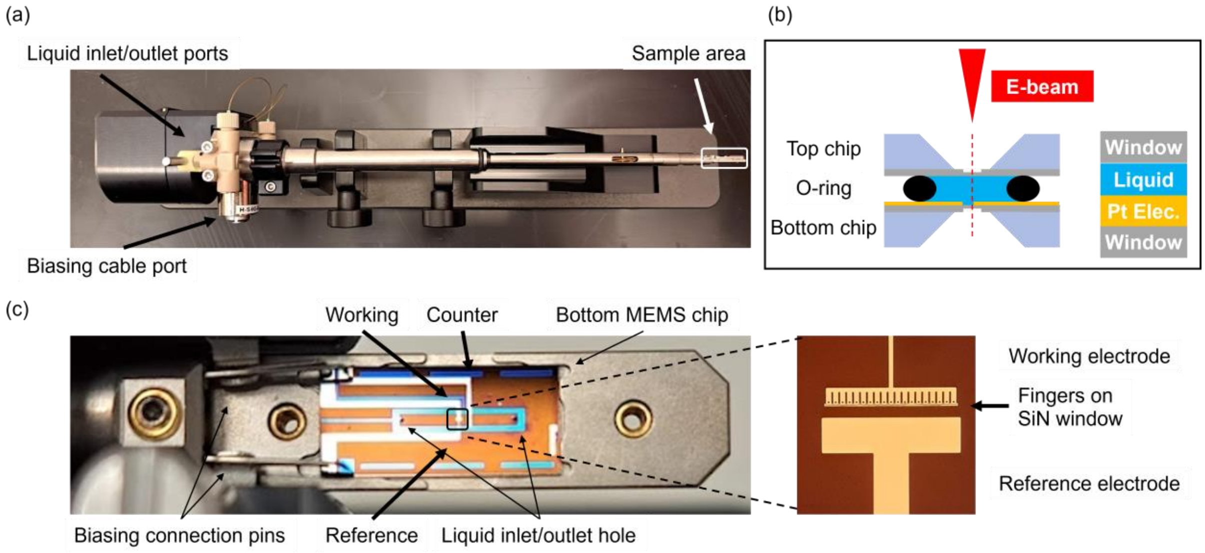

Figure S1: (a) Photograph of in situ liquid phase TEM holder highlighting the liquid inlet/outlet ports, biasing cable port and tip of the holder. (b) Schematic representation of the liquid cell. The on chip flow (not shown) allows liquid insertion within the sandwich MEMS chips. (c) Photograph of the tip of the holder with only the bottom MEMS chip. The bottom chip consist of Pt working, counter, and reference electrodes. The magnified image on the right side shows finger-shaped working electrode. The field of view during in situ TEM experiments covers only one of these fingers.

Pt vs Ag/AgCl

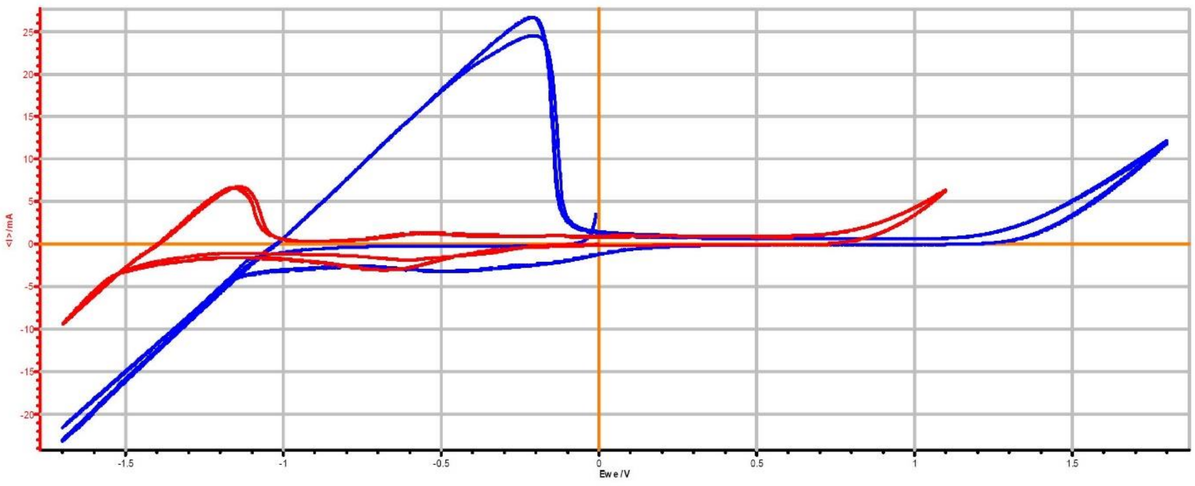

Figure S2: Shows the CV plots with 0.1M $ZnSO_4$ electrolyte at a scan rate of 0.01V/s in a typical beaker cell. Pt meshes are used as working as well as counter electrodes. The blue colored curves are obtained with Ag/AgCl as reference electrode while the red curves are obtained when another Pt mesh is used as the reference electrode. From this result Pt vs. Ag/AgCl is derived as 0.87V which can be used to get a rough estimate on Zn oxidation/ reduction potential. Geometrical differences between the Pt mesh electrode and the Pt microelectrode on the MEMS chips may corresponds to additional potential change.

Image processing: Segmentation of the plated Zn

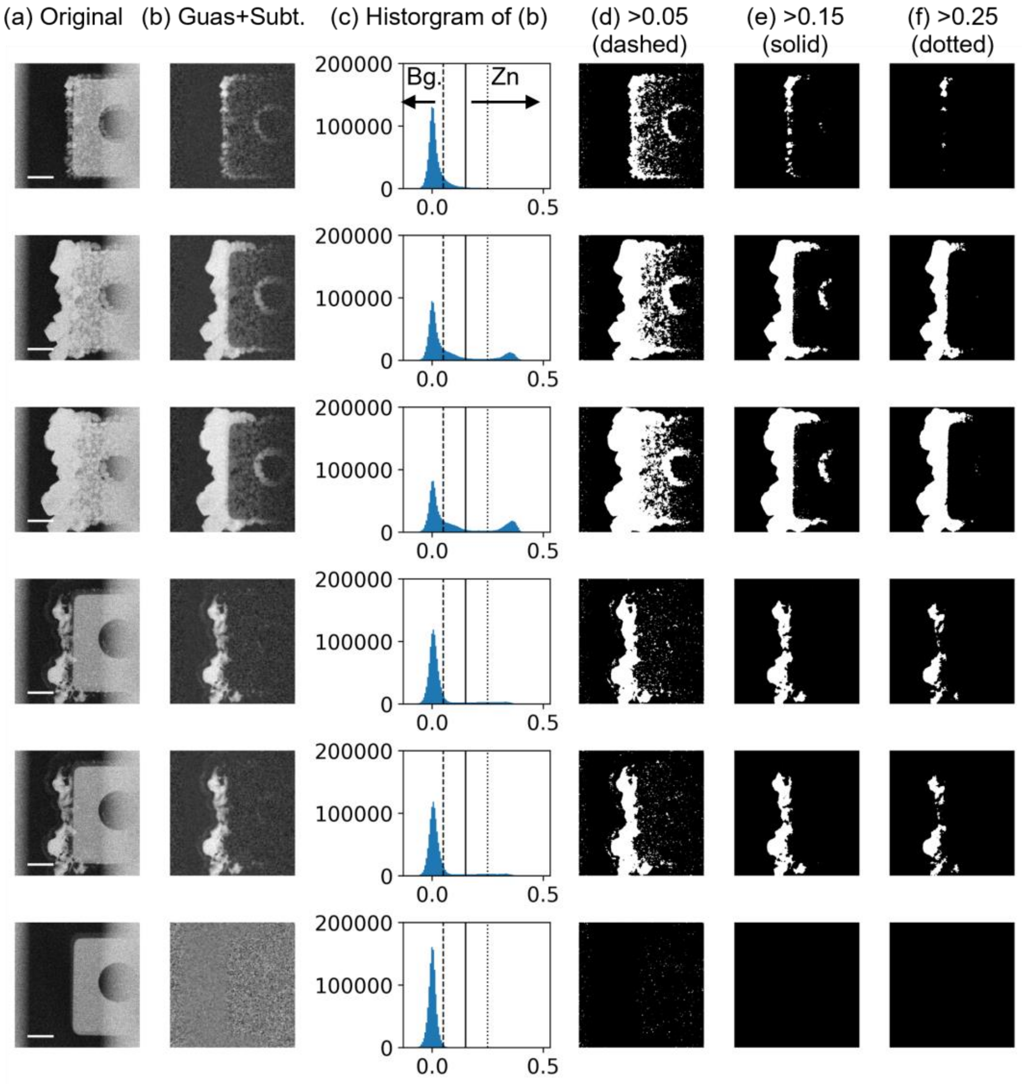

Figure S3: Scheme of image processing to segment the plated Zn: (a) Accquired STEM images. The scale bars is 2 µm. (b) Subtracted image from reference image after applying Gaussian filter. (c) Histogram (Count vs. Intensity) of (b). The inset lines indicate threshold candidate values (dashed: 0.05, straight: 0.15, and dotted: 0.25). (d-f) Binary segmented image by thresholding via corresponding values. Area with lower intensity becomes background area (black, SiN window and Pt electrode) and area with higher intensity becomes feature area (white, plated Zn). Threshold value was determined to 0.15 as proper value in this paper, because results from 0.05 contain features on top of Pt electrode, which misleads the result due to saturation issue written in Figure S5, and results from 0.25 contains shrinkage features at stripping cases (especially thinner area), which underestimates the result.

Image processing: 3D projection of the plated Zn

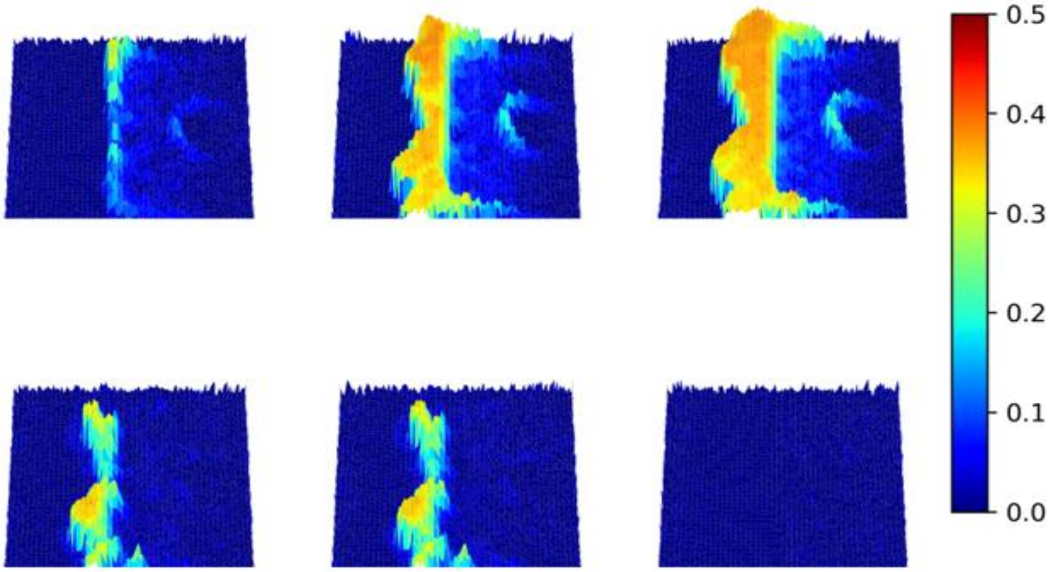

Figure S4: (a-f) 3D projected images of the Zn plating (a-c, at -1.2 V, -1.5 V, and -1.2 V) and stripping (d-f, at -0.5 V, 0.0 V, and 0.8 V) process after the Gaussian and subtraction process and utilizing the mass thickness contrast of high angle annular dark field STEM images.

STEM Detector saturation issue

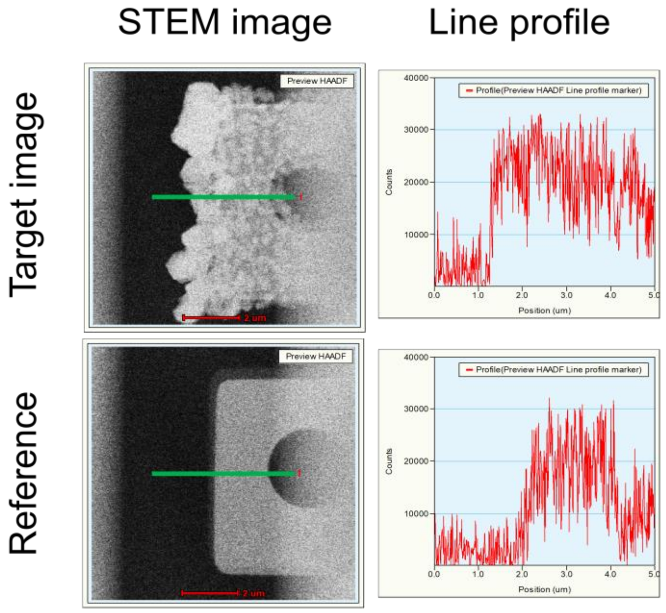

Figure S5: The line profiles in the reference and target image shows due to saturation of the detector, the plated Zn on top of the Pt electrode area cannot be quantified and even the plated Zn outside of the Pt electrode may be underestimated.

Image processing: Calibration of Z-intensity (mass thickness contrast) into length scale

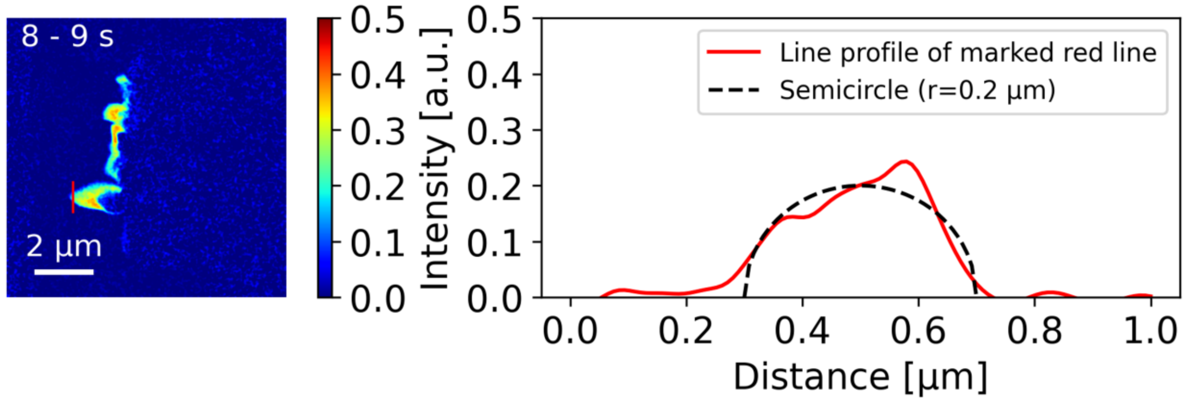

Figure S6: Shows the correctness of assuming a semi-cylindrical shape of the Zn dendrite growth to overcome the detector saturation issue for the intensity-thickness calibration.

Image processing: 4D STEM data processing

4D STEM data analysis was performed via python based LiberTEM package for both virtual ring detector and radial Fourier analysis methods.

1) Virtual ring detector

Virtual rings with the following inner and outer diameter was used for direct reconstruction of images from electron diffraction patterns using the ring analysis function in LiberTEM. Standard deviation of the electron diffraction pattern was tackled using the fluctuation EM function in LiberTEM.

- Inner ring: (0 – 0.1 nm^{-1})
- Outer ring: (9.1 – 14.0 nm^{-1})
- Zn (100 & 101): (4.1 – 5.0 nm^{-1})
- Zn (102): (5.3 – 6.4 nm^{-1})
- Zn (103 & 110): (6.9 – 7.8 nm^{-1})

2) Radial Fourier analysis

The steps of radial Fourier analysis is described in Figure S7. The detailed description of the method can be found in "4D STEM data processing via python" by A. Clausen et al., 'LiberTEM/LiberTEM: 0.9.2', https://doi.org/10.5281/zenodo.1477847

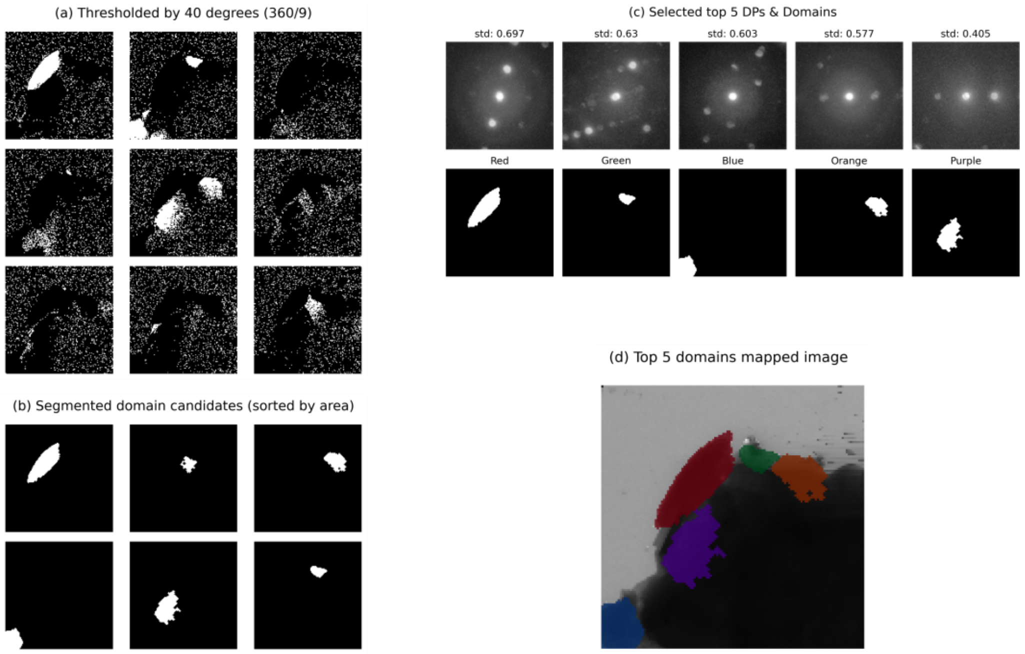

Figure S7: Scheme of orientation mapping by radial Fourier analysis method. (a) Thresholding with intensity (angle) by 9 steps (each 40 degrees) after radial Fourier analysis. (b) Segmenting the domain candidates with minimum area size. (c) Selecting the top 5 diffraction patterns and corresponding domains based on the standard deviation value of the reconstructed diffraction pattern. (d) Plotting top 5 domains on the virtual dark field image. The written color on each domain at (c) matches with (d).

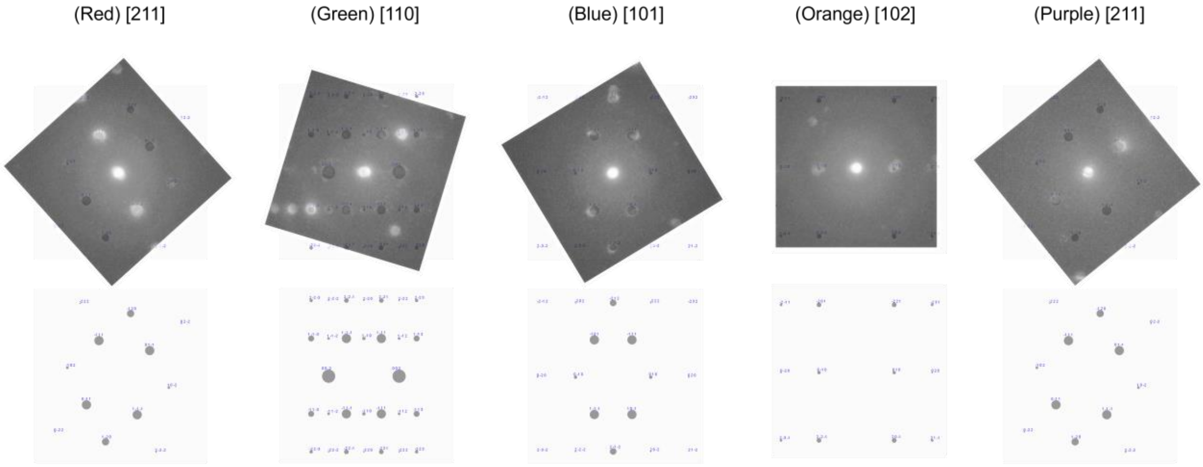

Figure S8: Comparison of top 5 reconstructed electron diffraction patterns with simulated electron diffraction pattern. Diffraction patterns were simulated via 'ReciprOgraph' software (https://www.epfl.ch/schools/sb/research/iphys/teaching/crystallography/reciprograph/) developed by N. Schoeni and G. Chapuis using Zn crystallographic structure (α=β=90°,

γ=120°, a=b=2.6648 Å, c=4.9467 Å) obtained from Crystallography Open Database (COD) (http://www.crystallography.net/) with id (9008522).

Supplementary movie captions

Movie S1. Synchronized video among HAADF STEM image, Current vs. Potential graph, Processed image and measured Zn deposition area of Zn plating and stripping experiment during cyclic voltammetry.
Movie S2. Synchronized video among HAADF STEM image and Processed image (within 1 second) of Zn dendritic growth during chronopotentiometry.
